# *Malus sieversii*: the origin, flavonoid synthesis mechanism, and breeding of red-skinned and red-fleshed apples

**DOI:** 10.1038/s41438-018-0084-4

**Published:** 2018-10-15

**Authors:** Nan Wang, Shenghui Jiang, Zongying Zhang, Hongcheng Fang, Haifeng Xu, Yicheng Wang, Xuesen Chen

**Affiliations:** 1 State Key Laboratory of Crop Biology, Shandong Agricultural University, Tai’an, 271018 Shandong China; 2 Collaborative Innovation Center of Fruit & Vegetable Quality and Efficient Production, Tai’an, 271000 Shandong China

## Abstract

Flavonoids play essential roles in human health. Apple (*Malus domestica* Borkh.), one of the most widely produced and economically important fruit crops in temperate regions, is a significant source of flavonoids in the human diet and is among the top nutritionally rated and most widely consumed fruits worldwide. Epidemiological studies have shown that the consumption of apples, which are rich in a variety of free and easily absorbable flavonoids, is associated with a decreased risk of various diseases. However, apple production is challenged by serious inbreeding problems. The narrowing of the hereditary base has resulted in apples with poor nutritional quality and low flavonoid contents. Recently, there have been advances in our understanding of the roles that *Malus sieversii* (Ledeb.) M.Roem has played in the process of apple domestication and breeding. In this study, we review the origin of cultivated apples and red-fleshed apples, and discuss the genetic diversity and construction of the core collections of *M. sieversii*. We also discuss current research progress and breeding programs on red-skinned and red-fleshed apples and summarize the exploitation and utilization of *M. sieversii* in the breeding of high-flavonoid, and red-fleshed apples. This study highlights a valuable pattern of horticultural crop breeding using wild germplasm resources. The future challenges and directions of research on the molecular mechanisms of flavonoid accumulation and high-flavonoid apple breeding are discussed.

## Molecular biology: Making apples even healthier

They say that an apple a day keeps the doctor away, but the breeding of new varieties of red-fleshed apples with higher flavonoid contents could make this widely-consumed fruit even healthier.

Apples are one of the richest dietary sources of flavonoids—metabolites credited with reducing the risk of various diseases—but during the process of domestication, the genetic diversity and nutritional quality of apples have decreased. In this review, Xue-Sen Chen at Shandong Agricultural University in China and colleagues track the evolution of modern cultivated apples from their ancestor, *Malus sieversii*, and outline recent developments in our understanding of the molecular pathways underpinning flavonoid synthesis. They also consider how *M. sieversii* could be used to create new varieties of tasty red-skinned and red-fleshed apples, enriched with beneficial metabolites.

## Introduction

Flavonoids are a major class of polyphenolic compounds produced by secondary metabolic pathways in plants^1^. They have been extensively studied for their essential roles in human health^1,2^. Flavonoids from various fruits and vegetables play a key role in reducing disease risk^3,4^. Apple (*Malus domestica* Borkh.), one of the most widely produced and economically important fruit crops in temperate regions^5^, is a significant source of flavonoids in the human diet and is among the top nutritionally rated and most widely consumed fruits worldwide^6^. There is a traditional American saying, “an apple a day keeps the doctor away.” In the US, 22% of the phenolics in the human diet originate from apples, which makes apples the largest dietary source of phenols^7^. In Spain, the median and mean total flavonoid intake were reported to be 313.26 and 269.17 mg/day, respectively, and apples are the largest source of dietary flavonoids, accounting for 23% of the total flavonoid intake^8^. In Finland, apples and onions are the primary sources of dietary flavonoids^9^.

The free flavonoid content is higher in apples than in other fruits, which results in the availability of more flavonoids for eventual absorption into the human bloodstream^10^. Apple flavonoid intake has been reported to have positive effects on aging and cognitive decline, cardiovascular health, weight management, asthma, and gastrointestinal health^11,12^. In addition, numerous epidemiological studies have found that apple consumption is widely associated with a decreased risk of various diseases^13,14^. However, the genetic diversity and the nutritional quality of modern apple cultivars have decreased during the process of domestication. Therefore, apple breeding with the target of enriching common apple cultivars with beneficial metabolites is a goal of both horticultural research and practice.

The exploration of the origin and evolution of apple and a greater understanding of the mechanisms of development of red-skinned and red-fleshed apples will enable researchers and breeders to cultivate superior and new varieties using different breeding objectives. In this review, we outline the historical domestication process of cultivated apples and red-fleshed apples, discuss recent research on the molecular mechanism of flavonoid synthesis and accumulation in red-skinned and red-fleshed apples, and summarize breeding goals and research progress on red-skinned and red-fleshed apples. We also discuss the exploitation and utilization of *Malus sieversii* (Ledeb.) M.Roem in the context of breeding for high-flavonoid and red-fleshed apple varieties. This information is significant to introduce wider diversity into apple breeding programs and highlights a valuable pattern of horticultural crop breeding.

## *Malus sieversii*: origin of cultivated apples and red-fleshed apples

### Origin of cultivated apples and red-fleshed apples

Apple is the primary fruit grown in temperate regions around the world^5^. To effectively utilize wild apple resources to breed apple hybrids, it is important to understand the origins of cultivated and red-fleshed apples, the relationship between cultivated apple and its primary wild resources, and the manner in which the key characters of apples have been domesticated.

Many researchers have focused on the origin and evolution of the cultivated apple. As early as 1930, the geneticist Vavilov considered that “the center of diversity is the center of origin” and speculated that the wild apple and its related species in Turkestan (Kazakstan, Kyrgyzstan, Uzbekistan, Turkmenistan, and Tajikistan) were the ancestors of the cultivated apple^15^. Later, Forsline et al. collected many wild germplasm resources in these regions, and their analyses appeared to confirm the similarity between wild and cultivated apples^16,17^. Concomitantly in 1996, Janick et al. suggested that “Central Asia” was the area of greatest diversity and the center of origin of the domesticated apple^18^. Genetic analyses based on random amplified polymorphic DNA (RAPD) data showed that *M. sieversii* from the Xinjiang Autonomous Region of China was the species most closely related to the cultivated apple *M. domestica* cv. “Golden Delicious”^19^. In addition, Rehder suggested that *Malus sylvestris* Miller could be one of the ancestors of the cultivated apple^20^. *Malus orientalis* Uglitz was proposed to be another ancestor of cultivated apple, but *M. sieversii* was considered to be the most important ancestor^21,22^.

**Fig. 1:**
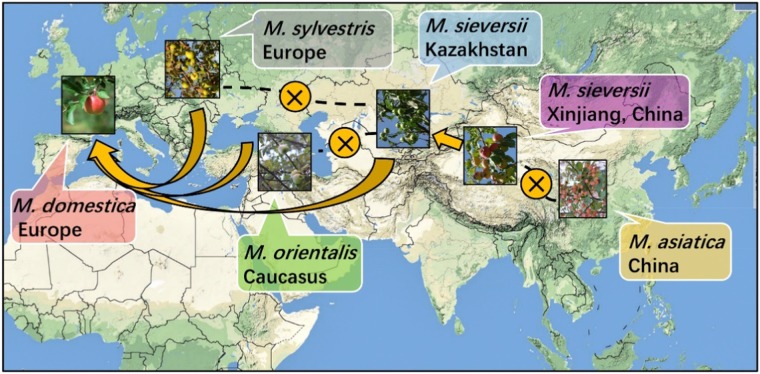
*Malus sieversii* in Central Asia, *Malus sylvestris* in Europe, and *Malus orientalis* in Caucasus were originally proposed to be ancestors of cultivated apple. Subsequent complete apple genome sequencing confirmed that the cultivated apple originated from *M. sieversii* in Central Asia, not *M. sylvestris* in Europe. *M. sieversii* in Xinjiang retains primitive characters and high homology, while *M. sieversii* in Kazakhstan has acquired genes from *M. sylvestris* and *M. orientalis* during the process of domestication into the cultivated apple. *M. sieversii* in Xinjiang moved eastward and crossed with *Malus baccata*, resulting in early Chinese apples

Until 2010, when the entire apple genome was first sequenced, new advances were developed in the origin and evolution of apples. Comparative analyses of genetic data showed that *M. domestica* cultivars were more closely related to accessions of the wild species *M. sieversii* and less closely related to accessions of *M. sylvestris*, *Malus baccata*, *Malus micromalus*, and *Malus prunifolia*^23^. This research confirmed that *M. sieversii* and not *M. sylvestris* was the ancestor of the cultivated apple. It was also confirmed that *M. orientalis* and *Malus asiatica* showed genetic similarity to *M. sieversii*. Subsequently in 2017, the genome re-sequencing of apple further clarified the evolutionary process of the cultivated apple. A population genetic structure analysis showed that both *M. domestica* cultivars and *M. sylvestris* originated from *M. sieversii*. *M. sieversii* in Xinjiang was found to be the most primitive and has retained high homology, while *M. sieversii* in Kazakhstan was found to have a high degree of genetic heterozygosity. *M. sieversii* migrated westward along the ancient Silk Road and gradually evolved into the cultivated common apple with the hybrid infiltration of *M. sylvestris* and *M. orientalis*. The crossing and domestication of *M. sieversii* and *M. baccata* along the Silk Road to the East resulted in early Chinese apples (Fig. 1)^5^.

Aside from cultivated apples, there are a number of high-anthocyanin and red-fleshed apple germplasm resources, and their origin and evolution have rarely been studied. Red-fleshed apples include *M. sieversii* f. *niedzwetzkyana* and the cultivated species *M. domestica* var. *niedzwetzkyana*^24^. Analyses of chloroplast and nuclear data showed that *M. domestica* var. *niedzwetzkyana* could have originated from the wild apple forests of Central Asia^25^. Steven, in 2012, identified and classified 3000 red-fleshed apple germplasm resources, including cultivars, wild species, and hybrids. It was inferred that all these red-fleshed apples originated from *M. sieversii* f*. niedzwetzkyana*^26^.

Some studies have shown that there is extremely rich genetic diversity related to polyphenol content, mineral elements, sugar and acid components, and volatile components in *M. sieversii* in Xinjiang^27,28^. The total phenolic and flavonoid contents were found to be significantly higher in *M. sieversii* than in the “Starking” apple variety^28^. Thus, *M. sieversii* can be used to breed functional apples with red flesh and high flavonoid contents. High-flavonoid apples will provide more flavonoids to consumers and will benefit human health.

### Genetic diversity of *Malus sieversii* and the construction of core collections

Studies on population genetic structure can provide the basis for in situ conservation and the utilization of germplasm resources^29^. The genetic structure refers to the non-random distribution of genetic variation in a species or population and indicates the distribution patterns of genetic variation within and between populations^30^. To elucidate the process, pattern, and mechanism of evolution, it is necessary to first discuss the genetic variation, genetic structure, and their variations within a population, as well as the factors that affect population genetic structure^31,32^.

An isozyme molecular variation analysis was conducted for 259 *M. sieversii* seedlings in Central Asia. The results showed that 85% of the isozyme variation was caused by differences among wild individual plants within the population, and the other 15% of variation was explained by differences among the sampling regions. There was no isozyme variation among populations in the same region^22^. Volk et al. analyzed the genetic diversity of 591 individual *M. sieversii* plants and found that most of the genetic differentiation was caused by differences among individual plants within the population^33^. In China, the Xinjiang wild fruit forest located east of the Tianshan Mountains is a rare “oceanic” broad-leaved forest type in the desert region. It is a remnant community of broad-leaved forest in this tertiary warm temperate zone, and it is distinct from the wild fruit forests in the west Tianshan Mountains of Central Asia^34^. *M. sieversii* in Xinjiang is a major component of the wild fruit forest in the Tianshan Mountains, and its genetic diversity is extremely rich^27,35^. Analyses of the peroxidase isozyme spectrum of *M. sieversii* in Xinjiang showed that there were clear differences among different *M. sieversii* apple populations but not within the same population^36^. When four *M. sieversii* populations in the Yili and Tacheng areas of Xinjiang were analyzed using simple sequence repeat and sequence-related amplified polymorphic markers, most of the genetic differentiation was within the population, and the genetic distance among the populations was significantly correlated with the geographic distance. The Gongliu population was the most genetically diverse of the populations tested and therefore had priority for protection^37,38^.

Although *M. sieversii* populations in Xinjiang have been severely damaged, they are still distributed across 1800 h m^2^, and there are nearly 1 million copies of the genetic resource^34^. The huge quantity of germplasm resources makes it very difficult to carry out ex-situ or in vitro conservation. In 1984, the concept of a core collection was proposed^39^. The construction of a core collection provides opportunities for the in-depth evaluation of the germplasm resources and the effective protection and utilization of those resources^40^. Marker-assisted sampling methods based on RAPD data were proposed^41^. Marshall and Brown suggested that the most important index to evaluate genetic diversity was the number of alleles^42^. Volk et al. surveyed and collected *M. sieversii* from Kazakhstan. Thirty-five of the 124 lines obtained were selected to construct the core collection^33^. Zhang et al. proposed a method to construct the core collection of *M. sieversii* in Xinjiang based on molecular markers^43^. Liu et al. suggested that the core collection of *M. sieversii* should be constructed based on the results of stepwise clustering, Mahalanobis distance, and single linkage analyses combined with preferred sampling (20% sampling proportion)^44^. The construction of the core collection of *M. sieversii* laid the foundation for its ex-situ conservation. To establish a multi-level conservation system for *M. sieversii* germplasm resources, research should focus on its ex-situ conservation, including the protection of its original habitat, and its preservation in vitro, either as plantlets or organs. These measures will be of great significance for the scientific protection and sustainable and efficient utilization of *M. sieversii* germplasm resources in apple breeding.

## Mechanisms regulating flavonoids in red-skinned and red-fleshed apples

**Fig. 2:**
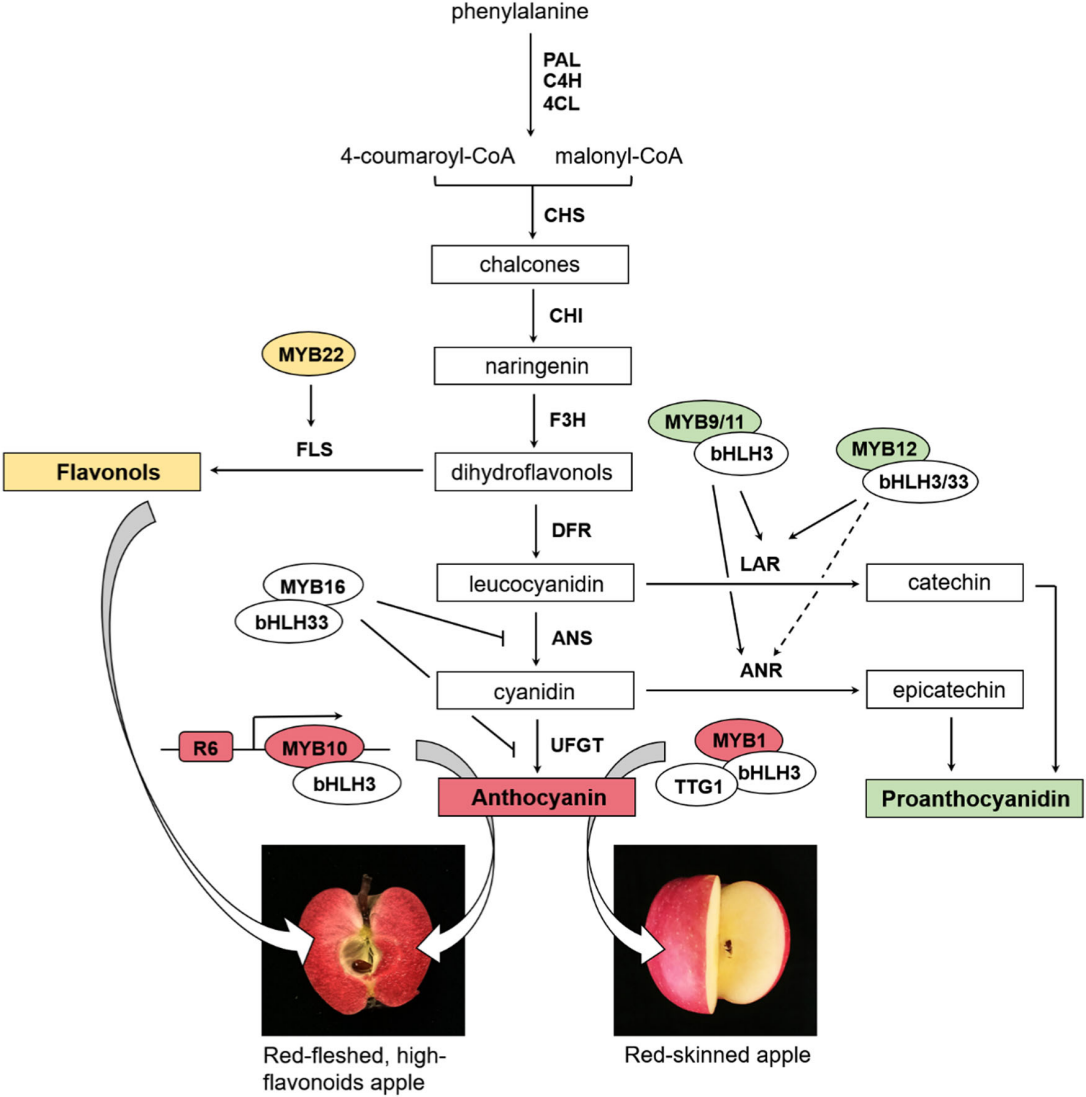
Simplified representation of the flavonoid biosynthetic pathway leading to the three major classes of end products: Flavonols, Proanthocyanidin, and Anthocyanin. PAL Phe ammonia lyase, C4H cinnamate-4-hydroxylase, 4CL 4-coumaroyl-CoA synthase, CHS chalcone synthase, CHI chalcone isomerase, F3H flavonoid 3-hydroxylase, FLS flavonol synthase, DFR dihydroflavonol-4-reductase, ANS anthocyanidin synthase, LAR leucoanthocyanidin reductase, ANR anthocyanidin reductase, UFGT UDP-Glc:flavonoid-3-O-glucosyltransferase

The complexity of the regulation mechanism of flavonoid biosynthesis is the embodiment of the diversity of apple germplasm resources. Therefore, in addition to understanding the origin and evolution of apple germplasm resources, it is highly significant to explore the metabolic mechanism of flavonoid production in apple. The pathways of flavonoid synthesis have been elucidated in many plant species^45–48^. Phenylalanine is a direct precursor for the synthesis of anthocyanins and other flavonoids, and it is converted by phenylalanine ammonia lyase (PAL) into 4-coumaroyl-CoA and malonyl-CoA^46,49^. The next key reaction is the conversion from 4-coumaroyl-CoA into dihydroflavonol, which can be converted into anthocyanins, flavonols and other flavonoids via the activities of different enzymes (Fig. 2).

### Flavonoid biosynthesis in red-skinned apple

The coloring of red-skinned apples is primarily determined by anthocyanin^47^. Anthocyanin biosynthesis involves the coordinated expression of structural genes and transcription factors^47^, and is influenced by other internal factors (endogenous hormones) and external factors (external environment, light, and temperature)^50^. Transcription factors determine the temporal and spatial patterns of gene expression and the expression level of structural genes, thus regulating the intensity and pattern of coloration in red-skinned apples. Members of three transcription factor families (MYB, bHLH, and WD40) function together in a ternary MYB–bHLH–WD40 (MBW) protein complex to participate in anthocyanidin pathways, a role that is widely conserved among plant species^51–53^. In the red-skinned apple, *MdMYB1* and *MdMYBA*, which regulate the biosynthesis of anthocyanin in fruit skin, were the first transcription factors to be isolated and characterized (Fig. 2)^47,54^. Since then, research on anthocyanidin synthesis in apple has progressed considerably. Ubiquitin ligase *MdCOP1* was suggested to interact with *MdMYB1* to regulate light-induced anthocyanin synthesis and apple skin coloration^55^. Subsequently, *MdMYB3* was also reported to promote anthocyanin accumulation in the apple skin^56^. *MdbHLH3* can bind to the promoters of *MdDFR* and *MdUFGT* and promote anthocyanin synthesis^57^. However, the transcription factor *MdTTG1* in the WD40 class does not bind to the promoters of *MdDFR* and *MdUFGT* or interact with *MdMYB1*. The regulation of anthocyanin by *MdTTG1* may be achieved by the interaction with *MdbHLH3* and *MdbHLH33*^58^.

In addition to gene regulation, anthocyanin synthesis in red-skinned apples is affected by light, temperature, and endogenous hormones. The expression of *MdMYBA* in the apple skin was induced by low temperature, and MdMYBA specifically combined with the promoter of *MdANS* to promote anthocyanin synthesis^54^. Low temperature induced the phosphorylation of *MdbHLH3* and enhanced its transcriptional activation activity on the promoter of the anthocyanin structural genes, resulting in the large-scale accumulation of anthocyanin^57^. However, high temperature was found to change the activity of the BMW complex and reduce anthocyanin accumulation, leading to lighter-colored apple skin^59^. *MdSnRK1.1* can interact with *MdJAZ18* to regulate the biosynthesis of anthocyanin induced by sucrose^60^. Jasmonic acid can increase the binding of *MdMYB9* and *MdMYB11* to the promoter of downstream structural genes in the anthocyanin metabolic pathway and promote anthocyanin accumulation^61^. In addition, studies have shown that plant nutrients also regulated the synthesis of anthocyanin^62^. For example, the redness of the apple skin can be influenced by the foliar application of CaCl_2_ and/or the nitrogen status^63,64^.

In conclusion, various transcription factors regulate anthocyanin synthesis via different mechanisms in red-skinned apple, and anthocyanin synthesis is also affected by the stage of growth and development and by environmental factors. Consumers prefer the appearance of red-skinned apples, and red apples have a higher commodity value^65^. However, apples are often consumed without the skin, and thus, the healthy flavonoids and anthocyanins are lost^66^. In addition, serious inbreeding problems have narrowed the hereditary base of cultivated apple varieties, resulting in fruit with poor nutritional quality and low flavonoid contents^67,68^. Tsao et al. analyzed eight apple cultivars and found that the flavonoid contents were five times higher in the peels than in the flesh^69^. Similarly, McGhie et al. found that, on average, 46% of the polyphenolics in whole apples were located in the skin, and the skin contained essentially all of the flavonols and anthocyanin^70^. However, consumers eat much smaller amounts of apple peels than flesh. Thus, the breeding of red-fleshed apples with high flavonoid content is a target of apple breeders worldwide. Although a few red-fleshed apple varieties have been bred, no high-quality commoditized red-fleshed apple varieties are available on the market at present. To produce new red-fleshed apple lines, wild red-fleshed apples and/or other apple resources are being used to carry out distant hybridization breeding. The cultivation of functional red-fleshed apple varieties is of great significance to expand the genetic basis of cultivated apple varieties and to increase the human intake of flavonoids to maintain health.

### Flavonoid biosynthesis in red-fleshed apple

The regulation of flavonoid synthesis in red-fleshed apples is more complex than that in red-skinned apples. Red-fleshed apples can be divided into Type 1 (red coloration in the fruit flesh, skin, leaves, and other vegetative tissues) and Type 2 (red coloration only in the fruit flesh). MdMYB10, an allele of *MdMYB1/MYBA* responsible for only the apple skin color, determines the red pigmentation of the Type 1 red-fleshed apple^71^. The Type 1 red-fleshed apple has a minisatellite-like structure comprising six tandem repeats in the promoter of *MdMYB10* (R6:*MdMYB10*), while white-fleshed apple has only one (R1:*MdMYB10*). The R6 repeat sequences are self-binding sites of the MdMYB10 protein and are positively correlated with the self-activating activity of its promoter (Fig. 2)^72^. The overexpression of *MdMYB10* in transgenic “Royal Gala” conferred a red-fleshed phenotype^73^. Although *MdMYB10* is highly expressed in Type 1 red-fleshed apples, it is not expressed in Type 2 red-fleshed apples. Instead, another MYB transcription factor close to *MdMYB10*, designated *MdMYB110a*, is correlated with the red pigmentation in Type 2 red-fleshed apples. *MdMYB110a* is not expressed in Type 1 red-fleshed apples^74^. These findings were confirmed in a study of the Type 2 red-fleshed apple “JPP35”^75^. Type 2 red-fleshed apples show more variation in the coverage and intensity of the red coloration of the cortex, indicating a more complex genetic control mechanism than that found in Type 1 red-fleshed apples^76^.

In addition to *MdMYB10*, other studies have also identified some positive and negative regulatory factors in the red-fleshed apple. For example, silencing *ANS* in a red apple cultivar almost completely blocked the biosynthesis of anthocyanin in the transgenic plants^77^. Wang et al. characterized a PA1-type MYB transcription factor, MdMYBPA1, from the red-fleshed apple and found that *MdMYBPA1* responded to low temperature by redirecting the flavonoid biosynthetic pathway from proanthocyanidin to anthocyanin production^78^. In addition, a transcriptomic analysis of the red-fleshed apples reveals the role of *MdWRKY11* in flavonoid and anthocyanin biosynthesis^79^. Cytokinin was suggested to promote the accumulation of anthocyanin by inhibiting the expression of *MdMYB308* in red-fleshed apples^80^. MdARF3 was induced by auxin to inhibit anthocyanin synthesis in red-fleshed apple calluses^81^. Xu et al. found that an anthocyanin negative regulatory gene, *MdMYB16*, may interact with *MdbHLH33* to control red pigmentation^82^. Another gene, *MdHB1*, has also been shown to negatively regulate anthocyanin synthesis. Its overexpression reduced the flesh content of anthocyanin in the red-fleshed apple “Ballerina”^83^. In addition to anthocyanin synthesis, there are few studies on the synthesis of proanthocyanidins and flavonols in red-fleshed apples. Recently, two novel R2R3-MYB transcription factors related to flavonoid synthesis, designated *MYB12* and *MYB22*, were cloned and identified from a red-fleshed apple. These transcription factors were demonstrated to play important roles in regulating the synthesis of proanthocyanidin and flavonols in red-fleshed apples (Fig. 2)^84^.

## Breeding of red-skinned and red-fleshed apples

### Breeding of red-skinned apples

With the increasing demand for higher quality apples, variety improvement has become an important goal for breeders^85^. Apple quality includes appearance, flavor, texture, storage and transportation properties, fresh food characteristics, suitability for processing, and genetic make-up^85^. In recent years, due to the rapid development of molecular biology technologies, there has been much progress in research on the mechanism of apple quality formation. To breed apples with improved flavor, Brown et al. and Visser et al. studied the classification standard of apple sugar and acid content, and the relationship between sugar and acid content and fruit flavor^86,87^. A recent study on the effect of SWEET genes on sugar accumulation in apple fruit provided the basis for the genetic improvement of flavor quality^88^. For texture quality breeding, researchers have targeted alleles related to ethylene, which plays an important role in climacteric fruits, such as tomato, apple, peach, and banana^89^. Ethylene production was found to be lower in the MdACS1-2/-2 homozygous variety than in the MdACS1-1/-2 heterozygous and MdACS1-1/-1 homozygous varieties^90^. To improve the processing qualities of apple, Huang et al. phenotyped hybrids derived from five biparental crosses of *M. asiatica* and *M. domestica* and demonstrated that relatively high acidity is an important breeding objective for fresh juice-specific apple cultivars^91^.

The red color of the skin is an important appearance trait of apple and largely determines its market value. Consumers always associate red skin with good taste, ripeness, and flavor; therefore, red-skinned apple varieties are preferred^65^. To breed red-skinned apples, identifying the genetic characteristics and regulation mechanism of red-skinned apples is the core issue. In early studies on the genetics and breeding of red-skinned apples, most breeders believed that the red color of the apple skin was a quality trait controlled by a dominant single gene. At the beginning of the last century, Crane et al. proposed for the first time that the synthesis of anthocyanin in the apple skin was controlled by the dominant single gene Rf, which was subsequently confirmed in other studies^92,93^. With the progress of biotechnology, the development of molecular markers has provided a theoretical basis for red-skinned apple breeding. Studies using molecular markers showed that yellow-skinned varieties were homozygous recessive, while red-skinned varieties were heterozygous or homozygous dominant^94,95^. Takos et al. obtained a MYB gene and a derived cleaved amplified polymorphic sequence marker in a study on the progenies of “Lady Williams” (red-skinned) and “Golden Delicious” (yellow-skinned)^47^. They found that MdMYB1-1 linked fragments could be amplified from most of the red cultivars, but none of the non-red cultivars.

In later studies, an increasing number of breeders suggested that the red-skin trait was controlled by multiple genes^96,97^. Lespinasse et al. analyzed the hybrid combinations of “Richared” and “Reinette di Landsberg” and found that the yellow-skinned trait was dominant to the red-skinned trait and was controlled by two dominant complementary genes. When there were two dominant genes, the fruit was yellow; otherwise, the fruit was red^96^. Other researchers suggested that the red-skinned trait was a quantitative trait controlled by many genes^24,98^. Sheng et al. proposed that the red-skinned trait was not only controlled by a dominant gene but also by the growth environment^99^. Ju et al. suggested that the red-skinned trait was controlled by a transcription factor and a structural gene, which participated in anthocyanin synthesis, and that environmental conditions affected skin color by regulating these genes^100^. Therefore, studies on the regulation of anthocyanin synthesis are highly significant to improve the appearance, quality, and commodity value of red-skinned apples.

It cannot be ignored that red sports selection is an important technique in red-skinned apple breeding. Approximately 30% of the apple varieties have been selected from apple sports, and their yields account for approximately half of the total apple production worldwide. Most of them are red sports^101,102^. Lines with changes in epigenetic modifications are known as sports, and sport selection can improve quality traits, such as the coloring, growth habit, and the timing of maturity in fruit trees. Epigenetic DNA methylation greatly affects anthocyanin synthesis and the coloration of the apple skin. Several studies have described the mechanisms of coloration in red sports. The increased anthocyanin synthesis in the red bud of “Ralls” was due to higher enzyme activities of CHI and DFR^103^. Additional research on the red bud of “Ralls” showed that the structural genes in anthocyanin biosynthesis were upregulated because of the increased expression of *MdMYB1*, and the low methylation level of the *MdMYB1* promoter was associated with its increased expression^104^. El-Sharkawy et al. suggested that differential methylation levels in the MR3 and MR7 promoter regions of *MdMYB10* were the epigenetic factors causing the color mutation^105^. Overall, the methylation level of the *MdMYB1* promoter determines apple color in color bud mutants. The formation of apple red buds is caused by the demethylation of the *MdMYB1* promoter. Additional research is required to identify the gene(s) involved in the demethylation process and how to recognize such loci.

### Breeding of red-fleshed apples

In the global apple industry, most of the significant commercial apple cultivars have white or off-white flesh, such as “Golden Delicious”, “Red Delicious”, “Fuji”, “Gala”, “Granny Smith”, and “Jonathan”. The use of a limited number of parents has greatly narrowed the genetic base of the existing cultivated apple varieties, which makes it difficult to obtain important breakthroughs in characters and phenotypes^67^. Thus, red-fleshed apples have attracted much attention from breeders because of their appealing flesh color.

The breeding of red-fleshed apple can be traced back to 1897 when Hansen encountered an unusual apple species in the wild fruit forest of Turkestan. This apple species had reddish-purple fruit skin, flesh, blossoms, and juvenile foliage. It was named *M. pumila* var. *niedzwetzkyana*, after its discoverer, the Russian botanist Niedzwetzky. He crossed *M. pumila* var. *niedzwetzkyana* with established varieties to create some new red-fleshed apples, the best known of which is “Almata”^106^. There is another pink-fleshed apple with a dull pale-green or whitish-yellow skin that is native to Siberia and the Caucasus. This apple, which is called “Surprise”, is widely thought to be a descendant of *M. pumila* var. *niedzwetskyana*^107^. After many years of apple breeding until 1944, Albert Etter produced approximately 30 high-quality red-fleshed descendants of “Surprise”, but only “Pink Pearl” was commercially released^108^. The Hansen apple shows deep red pigmentation not only in the apple flesh but also in the fruit skin, blossom, and juvenile foliage. This was later defined as the Type 1 phenotype^76^. The Etter apple only has pink flesh in the cortex, and the red pigmentation is not found in the leaves, stems, or other vegetative tissues. This was later defined as the Type 2 phenotype^109^. The pioneering work of Hansen and Etter in creating the two types of red-fleshed apple laid the foundation for the breeding of red-fleshed apple worldwide. In England, Fishman gave new names to Etter’s pink-fleshed apples; they were marketed under the “Rosetta” series title from 1973 and included “Pink Pearmain”, “Blush Rosette”, “Thornberry”, “Rubaiyat”, “Christmas Pink”, “Grenadine”, and “Pink Parfait”^108^. In Japan, Sekido et al. have been breeding Type 2 red-fleshed apples since 1989. The red-fleshed apple cultivar “Pink Pearl” and its progeny “JPP35” (“Jonathan” × “Pink Pearl”) were used as paternal parents to produce new red-fleshed cultivars^109^. In Germany, the Type 2 red-fleshed apple “Weirouge” was bred in Weihenstephan and first registered in 1997^110^. “Baya Marisa” was then bred using “Weirouge” as a parent. In New Zealand, the HortResearch apple breeding program, which was established in 1998, used the Type 1 red-fleshed cultivated apple “Redfield”, derived from “Wolf River” × *M. pumila* var*. niedzwetzkyana*^111^, for Type 1 breeding. For Type 2 breeding, descendants of “Sangrado” were used as the primary red-fleshed parents^75^. In Switzerland, a series of several red-fleshed apple cultivars, “Redlove”, have been bred and released by Markus Kobelt over the past decade, including “Redlove Sirena”, “Redlove Calypso”, “Redlove Circe”, “Redlove Era”, and “Redlove Odysso”^112^.

The German breeders Neumüller and Dittrich investigated apple seedlings originating from more than 40 cross-combinations to breed Type 1 red-fleshed apples. They found that a greater proportion of the red-fleshed progeny was obtained when the female parent was a Type 1 red-fleshed apple than when the male parent was a Type 1 red-fleshed apple^113^. In Japan, the breeding of the red-fleshed apple has primarily concentrated on the Type 2 red-fleshed apple, and the linkage between red-flesh traits and S-genotypes have been investigated. The S3-RNase allele in the red-fleshed apple cultivar “Pink Pearl” and its progeny, “JPP35”, were found to be linked with their red-flesh trait, which provided suitable cultivar combinations for efficient red-fleshed apple breeding^114,115^. In addition, to breed new red-fleshed apples containing both the *MdMYB10* and *MdMYB110a* genes, “Geneva” (Type 1) and “Pink Pearl” (Type 2) were crossed as paternal parents^116^. More than 3000 red-fleshed apple germplasm resources have been identified worldwide, and almost all the red types were found to the contain the R6:*MdMYB10* locus with the exception of “Pink Pearl”. Of the 82 non-red types, 76 contained only the R1:*MdMYB10* locus^26^. These findings provide additional evidence that the genetic background of red-fleshed apple is complex, and the mutation of any structural gene or transcription factor may affect the red-flesh phenotype.

### Utilization of *Malus sieversii* in red-fleshed apple breeding

*M. sieversii* belongs to the Tertiary relict species^34^. There are many variations in its fruit morphology, color, and flavor, plant height, tree shape, and other phenotypic characters. Thus, it is a critical genetic resource for apple breeding^117^. In addition, *M. sieversii* populations have developed resistance to drought, cold, pests, and barren soils under natural selection^118^, and therefore, this species has been widely used in breeding programs to improve the stress resistance of cultivated apples. In a study on the drought tolerance of six apple species, including *M. sieversii*, *Malus toringoides* was the most drought-resistant species followed by *M. sieversii* and *Malus transitoria*^119^. Twelve putative *M. sieversii* Hsp20 genes were identified from RNA-Seq data, indicating that the heat tolerance of *M. sieversii* may be associated with Hsp20^120^. In a study on biotic stress resistance, *M. sieversii* was found to be resistant to scab, and therefore, it has been used as a source of scab resistance in cultivar breeding^121,122^. Luby et al. evaluated the resistance of different provenances to fire blight, and found that provenances of *M. sieversii* had better resistance to fire blight and were suitable materials to breed for disease resistance^123^.

While *M. sieversii* has been used widely to breed for disease resistance, it has been less commonly used to breed for red-fleshed apples in recent years. Instead, some cultivated apples with good flavor quality have been used more widely for red-fleshed apple breeding. Although the early breeding of red-fleshed apples used *M. sieversii* and its descendants, the recent objectives of more colorful red flesh, better crispness, and better flavor have led to other lines being favored for red-fleshed apple breeding. Consequently, the other favorable characteristics of *M. sieversii*, such as its high-flavonoid content, short juvenile phase, and other excellent wild characteristics, have been neglected in the recent history of red-fleshed apple breeding.

Red-fleshed apples are not necessarily rich in flavonoids. In addition to anthocyanin, the primary pigment in red flesh, other flavonoid components are highly significant to the value of apples to human health. When selecting red-fleshed apples, breeders should monitor the changes in other flavonoids during the selection process^124^. Balancing the red-flesh phenotype with a high flavonoid content is the key to the effective utilization of *M. sieversii* f. *niedzwetzkyana*. Chen et al. directly used *M. sieversii* f. *niedzwetzkyana* as parents to breed “high-flavonoid functional apples”, which are defined as “apples that are rich in flavonoids in their red-fleshed fruits, that have a good quality of taste and flavor, and have a good quality of appearance and storage”^67^. In 2006, the F_1_ hybrid population of *M. sieversii* f. *niedzwetzkyana* was constructed in China^125^. Xu et al. determined the flavonoid composition and contents of four red-fleshed apple strains in the F_1_ hybrid population and suggested that the differential expression of *MdMYB10*, *MdbHLH3*, *MdMYB12*, *MdMYB16*, and *MdMYB111* at different stages of development could explain the differences in flavonoid contents^126^. The phenolic compounds that accumulated in five Type 2 red-fleshed apples with marked differences in the coverage and intensity of the red coloration of the cortex were also characterized. The results suggested that *MdMYB110a* could regulate the synthesis of other phenolic compounds, as well as anthocyanin^127^. Wang et al. conducted a comparative transcriptome analysis between red- and white-fleshed apples in the F_1_ population using RNA-Seq and found that 22 upregulated genes in red-fleshed apples were associated with flavonoid biosynthesis^128^.

Conventional hybrid breeding has remained the primary breeding method throughout the breeding process of the red-fleshed apple. Although hybrid breeding is an effective method to create new apple varieties, the long juvenile phase restricts its development. Therefore, shortening the juvenile phase has become an important research topic in apple breeding, and there have been some breakthroughs in recent years^129–131^. Flachowsky et al. transferred the flower-forming gene of *Betula pendula BpMADS4* into the apple cultivar “Pinova” and obtained an early flowering phenotype^101^. The first rapid and efficient breeding system for apple disease resistance was established using the *BpMADS4* transgenic line “T1190”, which shortened the breeding period from 30–40 years to several years^102^. Yamagishi et al. inoculated virus vectors containing the *Arabidopsis* flower-forming gene *AtFT* under the control of a strong promoter and the knock-down apple gene *MdTFL1-1* into the cotyledons of apple seedlings. Most of the seedlings bloomed, bore fruit, and produced seeds normally that year^103^. Nocker et al. suggested that grafting high-node buds onto dwarf rootstocks could shorten the juvenile phase^132^. However, such methods are labor-intensive and not suitable for large numbers of seedlings in a selection plot. The length of the juvenile phase of the hybrid offspring differs significantly among different apple varieties^133^. Chen et al. observed that the first filial generation has a short juvenile phase^67^. The juvenile phase of the five cross-combinations with *M. sieversii* f. *niedzwetzkyana* as parents was only 2.33–4.33 years, while that of the control hybrid combination of “Golden Delicious” and “Hanfu” was 3.33–5.33 years. This indicated that the utilization of *M. sieversii* f. *niedzwetzkyana* was useful to shorten the juvenile phase and improve the breeding efficiency of red-fleshed apples^68^.

The genome resequencing of *M. sieversii* has been completed. In addition, three complementary approaches to bridge the gap between genomics and breeding have been proposed^134^. It is expected that more excellent traits of *M. sieversii* f. *niedzwetzkyana* germplasm resources will be explored and utilized for red-fleshed apple breeding.

## Conclusions and perspectives

The complexity of the regulation of flavonoid biosynthesis is the embodiment of the diversity of red-fleshed and high-flavonoid apple germplasm resources. Therefore, research on the formation of the unique flavonoid profiles of red-fleshed apples is of great significance. Flavonoid metabolism in red-fleshed apples is affected by their genetic background and by environmental factors. Many genes in the flavonoid biosynthetic pathway have been cloned, identified, and partially functionally verified. However, due to the complexity of the metabolic pathway, additional research is required to explore the roles of other regulation mechanisms, such as protein modifications and small RNAs, in anthocyanin and flavonoid synthesis in red-fleshed apples.

The breeding of high-flavonoid red-fleshed apples involves the effective integration and balancing of multiple quality traits. For example, higher flavonoid contents in fruit can decrease fruit quality by creating a more astringent taste. Similarly, an increase in the anthocyanin content can lead to the accumulation of malic acid. Improving high-flavonoid apples by crossing them with existing cultivars with high sweetness, excellent flavor, high crispness, and other extreme phenotypes is the key focus in the next step of breeding. In addition, the more excellent traits of the *M. sieversii* f*. niedzwetzkyana* germplasm resources need to be explored in more detail and utilized to breed red-fleshed apples and early maturing or late-ripening red-fleshed high-flavonoid apple lines with excellent storage and transportation qualities.
